# A Case Study of Dysfunctional Nicotinamide Metabolism in a 20-Year-Old Male

**DOI:** 10.3390/metabo13030399

**Published:** 2023-03-08

**Authors:** Karen L. DeBalsi, John H. Newman, Laura J. Sommerville, John A. Phillips, Rizwan Hamid, Joy Cogan, Joshua P. Fessel, Anne M. Evans, Undiagnosed Diseases Network, Adam D. Kennedy

**Affiliations:** 1 Metabolon, Inc., Morrisville, NC 27560, USA; 2 Vanderbilt University Medical Center, Nashville, TN 37235, USA; 3 National Institutes of Health, National Center for Advancing Translational Sciences, Bethesda, MD 20892, USA

**Keywords:** NADH metabolism, nicotinamide *N*-methyltransferase, NADH deficiency, nicotinamide *N*-methyltransferase deficiency, nicotinamide, errors of metabolism

## Abstract

We present a case study of a 20-year-old male with an unknown neurodegenerative disease who was referred to the Undiagnosed Diseases Network Vanderbilt Medical Center site. A previous metabolic panel showed that the patient had a critical deficiency in nicotinamide intermediates that are generated during the biosynthesis of NAD(H). We followed up on these findings by evaluating the patient’s ability to metabolize nicotinamide. We performed a global metabolic profiling analysis of plasma samples that were collected: (1) under normal fed conditions (baseline), (2) after the patient had fasted, and (3) after he was challenged with a 500 mg nasogastric tube bolus of nicotinamide following the fast. Our findings showed that the patient’s nicotinamide N-methyltransferase (NNMT), a key enzyme in NAD(H) biosynthesis and methionine metabolism, was not functional under normal fed or fasting conditions but was restored in response to the nicotinamide challenge. Altered levels of metabolites situated downstream of NNMT and in neighboring biochemical pathways provided further evidence of a baseline defect in NNMT activity. To date, this is the only report of a critical defect in NNMT activity manifesting in adulthood and leading to neurodegenerative disease. Altogether, this study serves as an important reference in the rare disease literature and also demonstrates the utility of metabolomics as a diagnostic tool for uncharacterized metabolic diseases.

## 1. Introduction

Nicotinamide adenine dinucleotide (NAD(H)) is a coenzyme that plays an essential functional role in many biological processes. It serves as a redox carrier and hydride donor in energy metabolism [1], as a co-substrate for several enzymes involved in DNA repair and gene expression [2,3,4,5], and as a nucleotide analog in DNA ligation and RNA capping [6,7]. Through these functions, NAD(H) and its metabolites impact energy metabolism, DNA repair, epigenetics, inflammation, and the stress response. 

The importance of NAD(H) in maintaining homeostasis is shown by the negative impacts that a decrease in NAD(H) levels has on human health. NAD(H) depletes naturally with age [8,9] and is thought to be an underlying cause of many age-related pathologies, including diabetes [10], non-alcoholic fatty liver disease [11], Alzheimer’s disease [12], and atherosclerosis [13]. Genetic deficiency of NAD(H), such as in the case of glutamine synthetase deficiency or mitochondrial myopathy, is associated with a poor prognosis unless treatment is initiated [14,15].

NAD(H) is synthesized through three different pathways: the *de novo*, Preiss-Handler, and salvage, which start with tryptophan, nicotinate, and nicotinamide, respectively. Normally, in mammalian cells, NAD(H) synthesis primarily occurs through the salvage pathway [16]. Here, nicotinamide is converted to either 1-methylnicotinamide by the enzyme nicotinamide *N*-methyltransferase (NNMT) or to nicotinamide mononucleotide (NMN) by nicotinamide phophoribosyltransferase (NAMPT). NMN is then conjugated to ATP and converted to NAD(H) by NMN adenylyltransferase (NMNAT). The salvage pathway is coupled with several NAD^+^-consuming enzymes, including poly (ADP-ribose) polymerases (PARPs), sirtuins, CD38, CD157, and SARM1 [17]. These enzymes consume NAD^+^ to catalyze several reactions, including ones that generate nicotinamide. With the generation of nicotinamide, the salvage pathway can start over again. 

Despite nearly a century’s worth of findings that have characterized the roles of NAD(H) and its metabolites in health and disease, our understanding remains incomplete. Here, we present a case study of a critically ill 20-year-old male with an undiagnosed neurodegenerative disease and abnormalities in nicotinamide metabolism. Having ruled out multiple diagnoses, his care team turned to untargeted metabolomics to obtain a global metabolic profile in hopes of identifying the underlying cause(s) of his illness. The findings of that study identified an error in the patient’s nicotinamide metabolism. While this discovery did not directly impact the patient’s care or lead to a conclusive diagnosis, it demonstrates the value of metabolomics in revealing deep phenotypic insight into the underlying pathology of rare metabolic diseases and justifies its continued use in diagnostic and precision medicine initiatives.

## 2. Case Study

**Figure 1:**
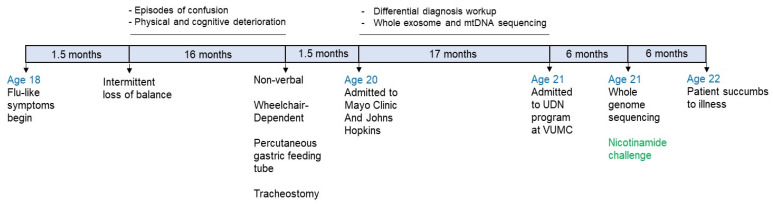
Timeline of the patient’s history.

The subject was a normal 18-year-old male college student who developed upper respiratory symptoms with nasal congestion, cough, non-specific headache, and mild fever. A nasal swab for influenza A was negative. No other family members were ill prior to or at the onset of the patient’s symptoms. The acute symptoms persisted for over two weeks. A chest radiograph and a CT sinus image were not informative. The acute illness regressed but never completely resolved. Approximately one and a half months after the onset of flu-like symptoms, the patient experienced intermittent loss of balance, which resulted in several falls. Over the next 16 months, he had periodic episodes of confusion and deteriorated to becoming non-verbal and wheelchair-dependent (Figure 1). One and a half years after the onset of the flu symptoms, he required a percutaneous gastric feeding tube and was given a tracheostomy with oxygen supplementation. 

Approximately 1.5 years after the flu symptoms appeared, he was admitted to the Mayo Clinic and then to Johns Hopkins Medical Center for study. The differential diagnosis was thought to include prion disease, paraneoplastic syndrome, inborn error(s) of metabolism, autoimmune encephalitis, and other genetic neurodegenerative diseases. His family history was negative for similar illnesses, and his personal history was negative for drug use, unusual exposures, psychiatric disorders, and trauma. He had traveled to Central America within the prior two years without illness.

A brain MRI showed only bilateral putamen T2 hyperintensities. An EEG showed moderate changes consistent with encephalopathy. His cerebrospinal fluid (CSF) showed elevated total Tau protein (2936 ng/mL) and was positive for 14-3-3, a clinical marker of Creutzfeldt-Jakob disease (CJD) [18,19]. However, an RT-QulC test ruled out CJD and other prion diseases. His CSF was also negative for numerous infections, including herpes simplex virus, Epstein-Barr virus, Varicella zoster virus, John Cunningham virus, enterovirus, West Nile virus, and human herpes virus 6. His CSF IgG and oligoclonal bands were normal, and cytology was negative for tumor cells. 

Immune studies ruled out autoimmune vasculitis, systemic lupus erythematosus, anti-*N*-methyl-d-aspartate (NMDA) encephalitis, and paraneoplastic syndrome. His blood levels of ceruloplasmin, copper, and complement C3 and C4 were also normal. His PET scan was normal. His electromyogram was non-specific and showed no evidence of peripheral neuropathy. A therapeutic trial of plasmapheresis conducted over a total of 5 sessions did not improve neurological function. 

Whole exosome and mitochondrial DNA sequencing were performed. These tests revealed maternally transmitted heterozygous variants of unknown significance (VUS) in the *RNASEH1* (RNASEH1 INM 002936.5:c.299G>A:p.Arg100His) and *LRSAM1* (NM_138361:c.375C>A:p.Asn125Lys), which encode proteins involved in DNA repair and cell adhesion, respectively. Single gene testing for Machado-Joseph syndrome and hexanucleotide repeats of *C9orf72* were negative. No rare genetic variants associated with encephalopathy were found. At this point, 3 years had passed since the onset of his flu-like symptoms. Given the extensive negative workup, he was referred to the Undiagnosed Diseases Network (UDN) program at Vanderbilt University Medical Center (VUMC). Due to the patient’s delicate health he did not travel to VUMC for his UDN visit. A telemedicine appointment was done, and all blood draws for diagnostic workups were performed locally. Whole genome sequencing (WGS) of both the patient and his parents did not reveal any shared or *de novo* candidate variants. The patient had a maternally inherited heterozygous VUS (NM 018210.3: c.805A>G:pArg269Gly) in *NAXD*, a gene involved in NAD(H) metabolism. Yet no second variant, either coding or non-coding, was found. 

A metabolic profile of the patient’s plasma revealed the absence of 1-methylnicotinamide and low *N*1-methyl-2-pyridone-5-carboxamide, two biochemical intermediates formed during the metabolism of nicotinamide into NAD(H) via the salvage pathway. Related metabolic compounds showed no obvious abnormalities, and his acylcarnitine and amino acid profiles were normal. A previous dose of NADH did not appear to have affected the patient’s condition. In attempts to further characterize the patient’s suspected defect in nicotinamide metabolism, one of the UDN physicians traveled to the patient’s home to perform a nicotinamide challenge. It had now been three and a half years since the patient first fell ill. By this time, he was completely bedridden with spastic reflexes and contractures. He required round-the-clock care, which his family provided to the highest degree of excellence. To perform the nicotinamide challenge, the patient was subjected to a 4-h fast and then immediately challenged with a 500 mg liquid bolus of nicotinamide. Plasma samples were collected: (1) under normal fed conditions, (2) immediately following the fast, and (3) 8 h after the challenge. Global metabolic profiling of those plasma samples revealed an error in metabolism related to nicotinamide *N*-methyltransferase (NNMT) activity. A reexamination of his WGS data did not show any candidate *NNMT* variants. Sadly, by the time this error of metabolism was discovered, the patient’s condition had deteriorated considerably. He died in March 2022, approximately four years after the onset of his illness. In the several hours leading up to his death, he experienced tachycardia to the 150–170 level, which gave way to cardiac arrest. There was no apparent sepsis, and the patient did not experience oxygen desaturation. Even though our findings did not lead to a conclusive diagnosis they did reveal metabolic defects that may have contributed, either directly or indirectly, to the patient’s complex disease. Our motivations for presenting the findings of this global profiling study are to ensure that this information will be available in the rare disease literature for future reference and to show the impact that metabolomics can have on characterizing rare metabolic diseases for both diagnostic and research purposes. 

## 3. Experimental Design

### 3.1. Sample Collection

**Figure 2:**
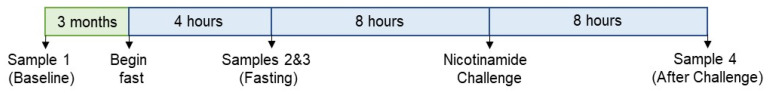
Sample collection time points.

As shown in Figure 2, the first whole blood sample (baseline) was collected from the patient under normal fed conditions three months before the metabolic profiling study was conducted. On the day preceding the study, the patient was subjected to a 4-h fast, and two paired whole blood samples were taken immediately following the fast. The next day, after the normal diet had resumed, the subject was given a 500 mg bolus of liquid nicotinamide through his gastric feeding tube to initiate nicotinamide metabolism. The last blood sample was taken 8 h after the challenge. All blood samples were collected into EDTA tubes by a trained phlebotomist. Plasma was isolated from whole blood by centrifugation and stored at −80 °C until analysis according to Metabolon’s sample handling protocol [20].

### 3.2. Sample Processing

Protein was precipitated from plasma by shaking with methanol on a SPEXC 2000 Geno/Grinder and centrifugation. For quality control (QC) purposes, several recovery standards were added to each sample before extraction. The extracted supernatants were divided into 4 aliquots and then placed on a sample evaporator (SPE-Dry 96) to remove organic solvents. Dried extracts were stored overnight under nitrogen. Solvent-only blanks were extracted using an identical method in every set to ensure curated biochemicals met a 3:1 signal-to-noise ratio. A plasma QC sample was extracted with 4 technical replicates in every set to monitor reproducibility. 

### 3.3. Ultrahigh Performance Liquid Chromatography-Tandem Mass Spectrometry (UPLC/MS-MS)

The patient’s global metabolic profile was resolved via untargeted ultrahigh-performance liquid chromatography-tandem mass spectrometry (UPLC/MS-MS) according to validated methods [21,22,23]. Briefly, all samples were subjected to four different chromatography methods. Each of the 4 aliquots of dried extracts were reconstituted in a solvent optimized for each method. Aliquot #1 was analyzed using acidic positive ion conditions optimized for hydrophilic compounds. The extract was gradient-eluted from a C18 column (Waters™ UPLC BEH C18-2.1 × 100 mm, 1.7 µm) using water and methanol containing 0.05% perfluoropentanoic acid (PFPA) and 0.1% formic acid (FA). Aliquot #2 was analyzed using acidic positive ion conditions optimized for hydrophobic compounds. The extract was gradient eluted from the same C18 column using methanol, acetonitrile, water, 0.5% PFPA, and 0.01% FA. Aliquot #3 was analyzed using basic negative ion-optimized conditions on a dedicated C18 column. The extract was eluted from the column with methanol, water, and 6.5 mM ammonium bicarbonate (pH 8.0). Aliquot #4 was analyzed using negative ionization after eluting from an HILIC column (Waters UPLC BEH Amide 2.1 × 150 mm, 1.7 um) using a gradient consisting of water and acetonitrile with 10 mM ammonium formate (pH 10.8). 

### 3.4. Compound Identification and Data Analysis

Compounds were identified by comparing the mass-to-charge (*m*/*z*), retention time, and associated fragmentation spectra in each sample to a library of standard chemical entities as described [21,22,23,24]. Technical replicates of plasma QC samples were extracted in each plate and interspersed throughout the run to monitor the analytical variability of biochemicals. All sample sets met our acceptance criteria of <10% relative standard deviation (RSD) for recovery standard variability and <15% RSD for instrument variability. Raw peak values from samples were used to derive the relative quantitation of each compound identified in the patient relative to a healthy reference population [25]. All values were log-transformed, then converted to *Z*-scores using rankit regression to estimate the mean and standard deviation as described in [26]. This analysis determined how many standard deviations the raw intensity of a given metabolite rose above or fell below the mean intensity of that metabolite in a dataset. Analyses were conducted in R [27] and Omicsoft Array Studio version 7.2. [28]. 

## 4. Results

### 4.1. Confirmation of Analytical Precision

To confirm an acceptable precision and accuracy of the metabolomics data, we analyzed 6 technical replicates of plasma. A total of 943 metabolites were detected, with 872 detected in all 6 replicates. Those 872 metabolites had a mean RSD of 9.04% and a median RSD of 3.7%. A total of 43 internal and recovery standards were analyzed to assess variability in metabolite recovery across instruments. The metabolites detected in those standards had a mean and median RSD of 5.1% and 4.1%, respectively, meeting Metabolon’s acceptance criteria for variability (see *Compound Identification and Data Analysis*).

### 4.2. Nicotinamide Metabolism

**Figure 3:**
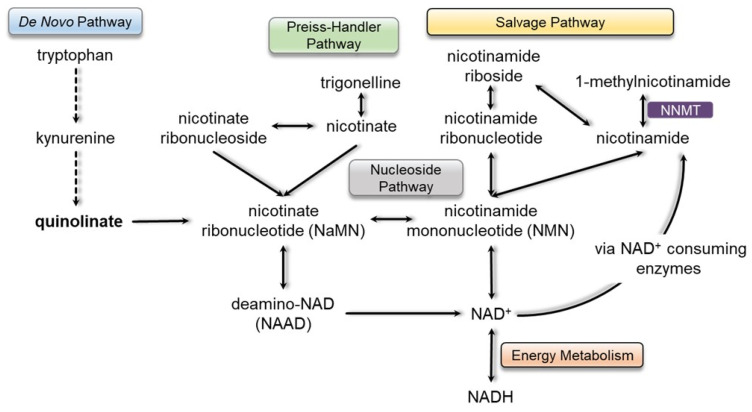
NAD(H) biosynthesis pathways.

**Figure 4:**
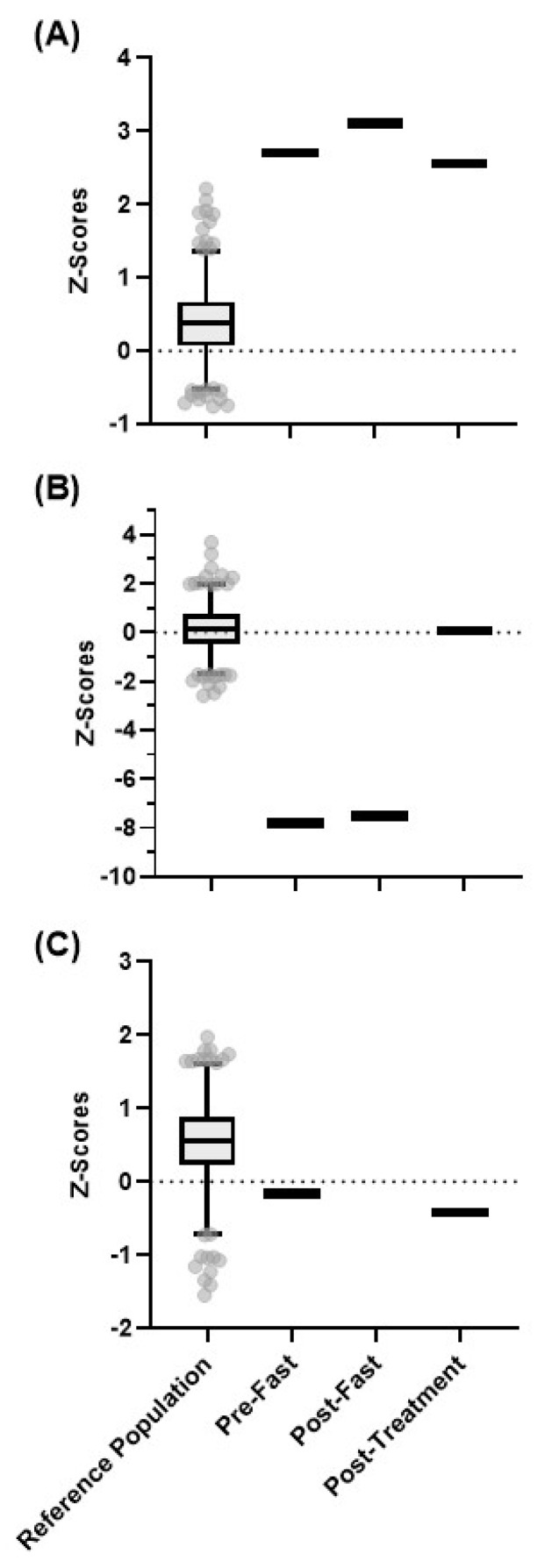
The *Z*-scores for the healthy reference population in comparison to the pre-fast, post-fast, and post-treatment *Z*-scores for the patient for (**A**) nicotinamide, (**B**) 1-methylnicotinamide, and (**C**) nicotinamide riboside. The box represents the 1st and 3rd quartiles, with the median indicated by a line within the box. The whiskers represent the top 97.5th and bottom 2.5th percentiles of the reference range. Individual values within the reference population (N = 866) outside these percentiles are indicated with circles.

Under physiologic conditions, the enzyme *N*-methyltransferase (NNMT) metabolizes nicotinamide to 1-methylnicotinamide (Figure 3). While the role of 1-methylnicotinamide in physiologic processes remains the subject of study, it is hypothesized to be a signaling molecule that plays a role in energy metabolism [29], coagulation [30], and T-cell activity in cancer [31]. A previous metabolic profile showed the patient had a total absence of 1-methylnicotimide even though he was receiving daily high-dose supplementation of nicotinamide via standard enteric nutrition. Therefore, to evaluate the patient’s ability to absorb and metabolize nicotinamide, we performed global, untargeted metabolic profiling of plasma collected at baseline 4 h after fasting and 8 h after being challenged with 500 mg of liquid nicotinamide (Figure 4). 

The patient’s nicotinamide levels were in the 100th percentile at all three time points, confirming the effect of high nicotinamide supplementation. The patient’s baseline and fasting levels of 1-methylnicotinamide were below the 2.5th percentile of the reference population, suggesting that his NNMT function was decreased. Surprisingly, the nicotinamide challenge restored his 1-methylnicotinamide levels to normal (Figure 4B), indicating that the patient had some functional NNMT, though its action was only induced by a large dose of nicotinamide following a fast. This finding also suggests that the metabolic defect at play favored the conversion of nicotinamide to nicotinamide riboside under baseline conditions. Interestingly, nicotinamide riboside levels were normal (40th percentile) at baseline and after fasting and rose only slightly (44th percentile) after the nicotinamide challenge (Figure 4C). While nicotinamide mononucleotide (NMN) was not detected at any time point, NAD^+^ was detected, but only at very low levels near the limit of detection. This is likely due to relatively low levels of both these intermediates in plasma. 

**Figure 5:**
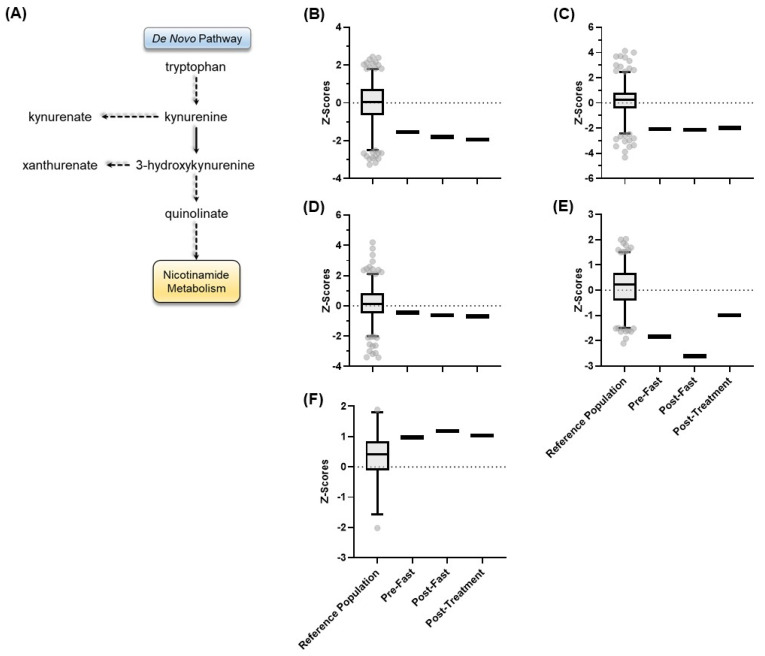
(**A**) The *de novo* pathway for the NAD^+^ biosynthesis, along with the *Z*-scores for the healthy reference population in comparison to the pre-fast, post-fast, and post-treatment *Z*-scores for the patient for (**B**) tryptophan, (**C**) kynurenine, (**D**) kynurenate, (**E**) xanthurenate, and (**F**) quinolinate. The box represents the 1st and 3rd quartiles, with the median indicated by a line within the box. The whiskers represent the top 97.5th and bottom 2.5th percentiles of the reference range. Individual values within the reference population (N = 866) outside these percentiles are indicated with circles.

We next examined metabolite levels in the *de novo* NAD(H) biosynthesis pathway (Figure 5A). Here, tryptophan is converted to kynurenine, which is then converted to quinolinate. Quinolinate goes through a series of reactions that result in the generation of NAD(H). Tryptophan, kynurenine, and quinolinate were all within the low normal range at all three time points (Figure 5B–F), suggesting that the patient’s metabolic defect was confined to the salvage NAD(H) biosynthesis pathway. 

### 4.3. Methionine Metabolism

**Figure 6:**
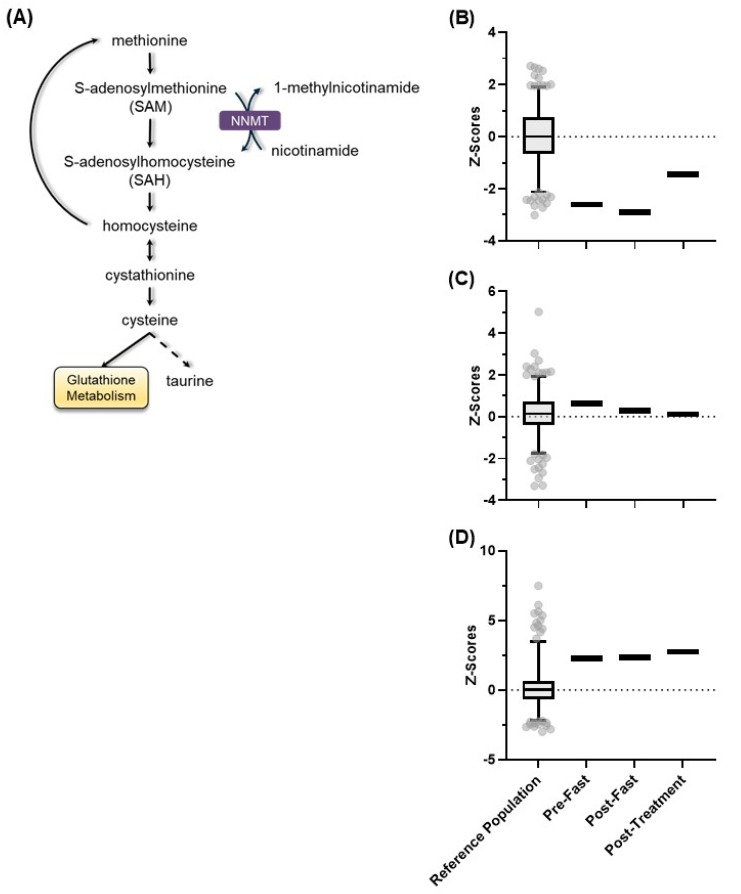
(**A**) Methionine metabolic pathway, along with the *Z*-scores for the healthy reference population in comparison to the pre-fast, post-fast, and post-treatment Z-scores for the patient for (**B**) methionine, (**C**) cystathionine, and (**D**) cysteine. The box represents the 1st and 3rd quartiles, with the median indicated by a line within the box. The whiskers represent the top 97.5th and bottom 2.5th percentiles of the reference range. Individual values within the reference population (N = 866) outside these percentiles are indicated with circles.

To better characterize the patient’s metabolic profile and further investigate the role of NNMT in his symptoms, we examined biochemicals in the methionine metabolism pathway. Methionine is an essential amino acid found in meat, fish, and dairy products. It is one of several precursors of cysteine, which is converted to glutathione in the tissues. Glutathione plays a vital role in antioxidant defense, nutrient metabolism, regulation of gene expression, DNA and protein synthesis, and cell proliferation. NNMT plays a role in methionine metabolism by converting the homocysteine precursor, *S*-adenosylmethionine (SAM), to *S*-adenosylhomocysteine (SAH). SAH is then converted to homocysteine, which is either converted back to methionine or cystathionine, leading to downstream generation of cystine and glutathione (Figure 6A).

Methionine was absent in the patient’s plasma at baseline and after fasting (Figure 6B), but the nicotinamide challenge restored methionine levels to the low normal range (7th percentile). This suggests that the reduced function of NNMT under baseline and fasting conditions impaired the conversion of SAM to SAH. This would then impair the conversion of homocysteine back to methionine. Interestingly, cystathionine, the metabolite directly downstream of homocysteine, was within the normal range at all three time points (Figure 6C). Cysteine, which is immediately downstream of cystathionine, was significantly elevated (*p* < 0.05) at all time points (Figure 6D). These data further suggest that NNMT function was minimal at baseline and that its function was restored in response to a nicotinamide bolus, resulting in a correction of metabolic pathways affected by low NNMT activity.

### 4.4. NAD(H) Production

**Figure 7:**
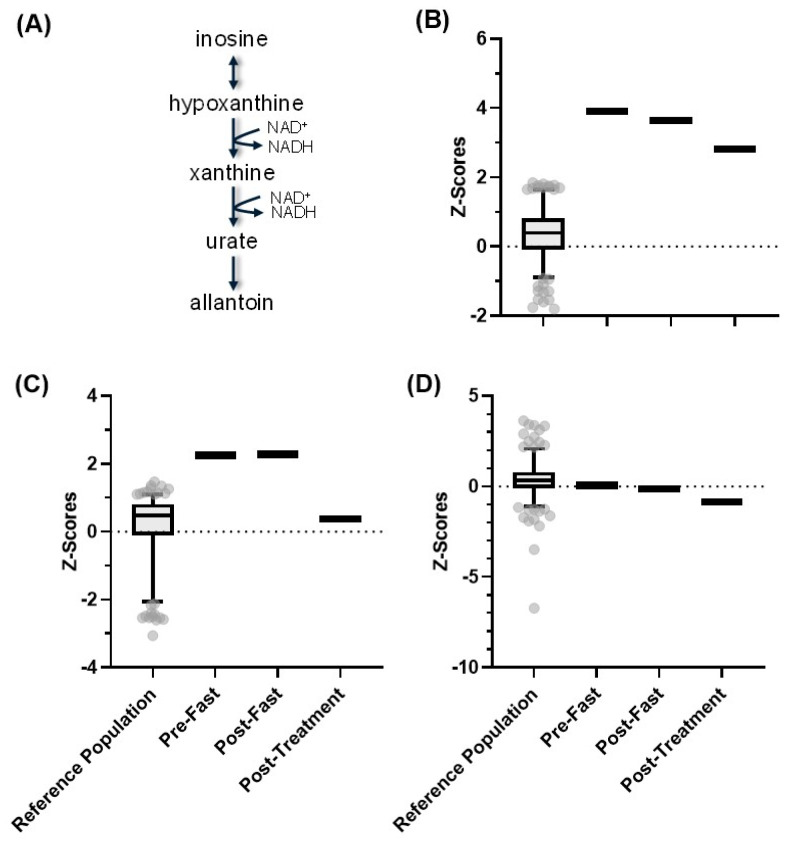
(**A**) Purine metabolic pathway (through inosine), along with the *Z*-scores for the healthy reference population in comparison to the pre-fast, post-fast, and post-treatment *Z*-scores for the patient for (**B**) inosine, (**C**) hypoxanthine, and (**D**) xanthine. The box represents the 1st and 3rd quartiles, with the median indicated by a line within the box. The whiskers represent the top 97.5th and bottom 2.5th percentiles of the reference range. Individual values within the reference population (N = 866) outside these percentiles are indicated with circles.

Since NAD(H) is difficult to profile directly, we investigated the patient’s ability to produce this molecule by examining purine metabolism pathways. Purine metabolism maintains cellular pools of adenylate and guanylate that are derived through the synthesis and degradation of purine nucleotides. In purine catabolism, inosine is converted to hypoxanthine via salvage pathway reactions. Hypoxanthine is converted to xanthine and then to urate through reactions that require the reduction of NAD^+^ to NADH (Figure 7A). At baseline and after fasting, the patient’s inosine and hypoxanthine levels were above the 97.5th percentile of the normal reference population (Figure 7B,C). The nicotinamide challenge brought hypoxanthine levels down to the normal range. Xanthine levels were within the normal range at all three time points but fell from the 47th percentile to the 19th percentile in response to the nicotinamide challenge (Figure 7D). While the change in xanthine was not significant, it, along with a reduction in hypoxanthine, suggests that this patient could make NAD(H) via nicotinamide metabolism.

### 4.5. Inflammatory Response

**Figure 8:**
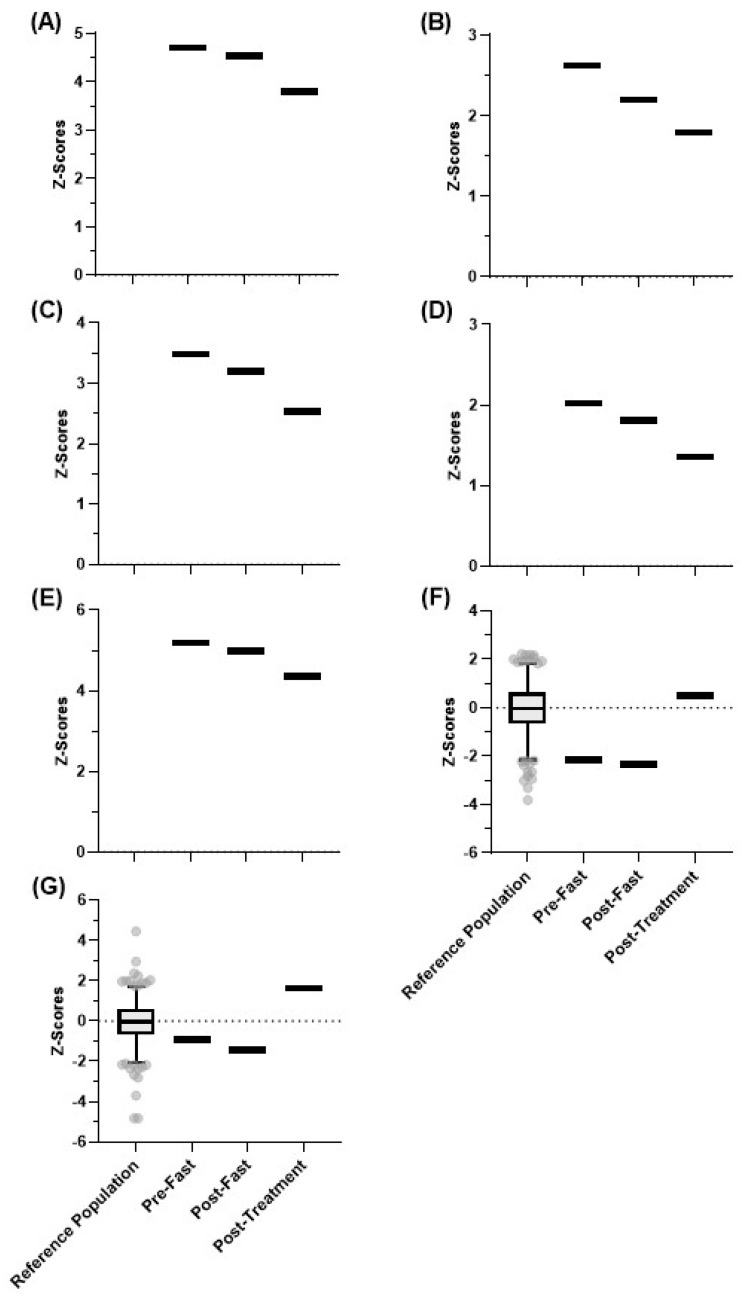
*Z*-scores for the healthy reference population in comparison to the pre-fast, post-fast, and post-treatment *Z*-scores for the patient for (**A**) stearidonate, (**B**) docohexaenoate, (**C**) docopentaenoate, (**D**) linolenate, (**E**) eicosapentaenoate, (**F**) cortisol, and (**G**) cortisone. The box represents the 1st and 3rd quartiles, with the median indicated by a line within the box. The whiskers represent the top 97.5th and bottom 2.5th percentiles of the reference range. Individual values within the reference population (N = 866) outside these percentiles are indicated with circles.

We observed a significant elevation in several polyunsaturated fatty acids (PUFAs) essential for generating lipid inflammatory mediators. PUFAs can be classified as n-3 or n-6 fatty acids based on their chemical structure. n-3 and n-6 fatty acids are ligands for the nuclear receptors NFκB, PPAR, and SREBP-1c, which regulate the expression of various genes involved in inflammatory signaling and lipid metabolism [32]. n-3 PUFAs tend to down-regulate the expression of inflammatory genes and the synthesis of lipids, while n-6 PUFAs tend to upregulate these functions [33,34]. Thus, the ratio of n-3 to n-6 PUFAs in the cells strongly impacts cellular processes, including cell death and survival. Here, we found that the n-3 PUFAs stearidonate, docosahexaenoate, docosapentaenoate, linolenate, and eicosapentaenoate were above the normal range at baseline and after fasting. Levels of stearidonate, docosapentaenoate, and eicosapentaenoate were above the normal range at baseline and after fasting (Figure 8A–E). We did not observe elevations in any n-6 PUFAs. Stearidonate, docosapentaenoate, and eicosapentaenoate levels remained above normal after the nicotinamide challenge, while docosahexaenoate and linolenate fell into the normal range. These data suggest that the patient’s inflammatory response was dampened under baseline conditions and was not significantly affected by the nicotinamide challenge. We note that the patient demonstrated a significant increase in the anti-inflammatory mediators cortisol and cortisone following the challenge. However, neither of these mediators rose above the normal reference range (Figure 8F–G).

## 5. Discussion

Nicotinamide *N*-methyltransferase (NNMT) is a cytosolic enzyme that catalyzes the conversion of nicotinamide to 1-methylnicotinamide in the salvage pathway of NAD(H) biosynthesis and the conversion of *S*-adenosylmethionine to *S*-adenosylhomocysteine in the methionine metabolic pathway. It actively mediates genome-wide epigenetic and transcriptional changes through hypomethylation of repressive chromatin marks. It is also a crucial player in the biosynthesis of NAD(H) molecules, and vital for normal energy metabolism, DNA repair, gene expression, and stress response [35,36]. 

Similarly to NAD(H), maintaining homeostatic levels of NNMT is considered important for preserving human health. Aberrantly high expression of NNMT has been linked to the development of obesity [37], type 2 diabetes [38], various cancers [39,40,41], neurodegenerative diseases, including Alzheimer’s and Parkinson’s disease [42,43,44], and pulmonary hypertension [45]. On the other hand, abnormally low serum levels of NNMT have been reported in bipolar mania patients [46], and higher incidents of familial schizophrenia have been correlated with lower expression of NNMT in the frontal cortex [47]. While aberrant expression of NNMT is associated with various diseases, the mechanisms through which it drives disease progression is an ongoing focus of investigation. For example, in cancer, NNMT is highly expressed, which is thought to facilitate dysfunctional metabolism leading to tumor growth [48]. Still, at present, there is a paucity of evidence to show that the dysfunction of NNMT activity itself is a key driver of disease. Undoubtedly, the role of NNMT in health and disease is complex, and our understanding of the mechanisms that govern its role remains to be fully understood. 

To our knowledge, this is the first report of a patient with decreased NNMT function that either manifested in or became apparent in adulthood. The only clue to the underlying cause of the patient’s disease was the absence of 1-methylnicotinamide and low *N*1-methyl-2-pyridone-5-carboxamide in his plasma, which was revealed by a metabolic profiling test performed prior to the study described herein. To further investigate a probable defect in nicotinamide metabolism, we performed untargeted global profiling of plasma samples collected at baseline, after fasting, and after receiving a 500 mg bolus of nicotinamide through his gastric feeding tube. 

Our findings revealed that the patient had severely low NNMT function, which was restored to normal in response to the nicotinamide challenge. Two metabolites that rely on NNMT for biosynthesis, 1-methylnicotinamide, and methionine, increased from undetectable levels to the normal range in response to the nicotinamide challenge, thus demonstrating the presence of functional NNMT (Figure 4 and Figure 6). Interestingly, while the nicotinamide challenge increased 1-methylnicotinamide levels from nil to normal, the amount of nicotinamide riboside increased only slightly, suggesting that the patient’s metabolic defect heavily favored the conversion of nicotinamide to nicotinamide riboside at baseline. No other overt defect in nicotinamide biosynthesis was found (Figure 5). Examination of purine metabolism showed a high accumulation of hypoxanthine and its direct downstream intermediate xanthine at baseline and after fasting. Hypoxanthine is converted to xanthine, which is then converted to urate by two reactions that require the oxidation of NADH to NAD^+^. The nicotinamide challenge increased the conversion of NADH to NAD^+^, as evidenced by the reduction of hypoxanthine and xanthine, suggesting that the patient could indeed biosynthesize NAD(H) (Figure 7). The patient exhibited higher than normal expression of inflammatory polyunsaturated fatty acids (PUFAs) and lower than normal expression of anti-inflammatory PUFAs. The nicotinamide challenge did not affect this expression profile (Figure 8). 

Altogether, our data demonstrated functional NAD(H) biosynthesis and a defect in NNMT function of unknown origin that could be rescued with a high dose of nicotinamide after fasting. Interestingly, rescuing NNMT function did not impact the patient’s health status. This suggests that either the patient’s condition had deteriorated to the point where treatment was no longer effective or that there was/were other metabolic defect(s) at play that were not captured in the plasma metabolome. One limitation of this study was the lack of metabolic profiling of muscle tissue where NAD(H) and other intermediates of nicotinamide are easier to detect. Profiling the patient’s CSF could have also provided further insight into his nicotinamide metabolism and potentially revealed other mechanisms to explain his symptoms. Finally, we note that in the absence of a conclusive genetic cause of the disease along with the age of disease onset, we cannot categorically say whether the patient’s error in nicotinamide metabolism was inborn or acquired. This case was highly complex, and our findings did reveal information about the patient’s metabolic state, which we hope will serve as a reference in the annals of the rare disease literature. Our findings show that untargeted metabolic profiling is a powerful tool for elucidating deep phenotypic information on individuals with inborn or acquired errors of metabolism and should continue to be used to aid diagnosis and advance our understanding of these diseases. 

## Data Availability

The data presented in this study are available in the main article.

## Supplementary Materials

The following supporting information can be downloaded at: https://www.mdpi.com/article/10.3390/metabo13030399/s1.
