# Supplementary material for: A Case Study of Dysfunctional Nicotinamide Metabolism in a 20-Year-Old Male
Source: Metabolites. 2023 Mar 8;13(3):399. doi: 10.3390/metabo13030399 (PMC10055858; doi:10.3390/metabo13030399)
Supplement: Supplementary file 1 [file metabolites-13-00399-s001.zip › metabolites-2223343-supplementary.pdf]

Members of the Undiagnosed Diseases Network

Maria T. Acosta
Margaret Adam
David R. Adams
Raquel L. Alvarez
Justin Alvey
Laura Amendola
Ashley Andrews
Euan A. Ashley
Carlos A. Bacino
Guney Bademci
Ashok Balasubramanyam
Dustin Baldridge
Jim Bale
Michael Bamshad
Deborah Barbouth
Pinar Bayrak-Toydemir
Anita Beck
Alan H. Beggs
Edward Behrens
Gill Bejerano
Hugo J. Bellen
Jimmy Bennett
Beverly Berg-Rood
Jonathan A. Bernstein
Gerard T. Berry
Anna Bican
Stephanie Bivona
Elizabeth Blue
John Bohnsack
Devon Bonner
Lorenzo Botto
Brenna Boyd
Lauren C. Briere
Gabrielle Brown
Elizabeth A. Burke
Lindsay C. Burrage
Manish J. Butte
Peter Byers
William E. Byrd
John Carey
Olveen Carrasquillo
Thomas Cassini
Ta Chen Peter Chang
Sirisak Chanprasert
Hsiao-Tuan Chao
Ivan Chinn
Gary D. Clark
Terra R. Coakley
Laurel A. Cobban

Joy D. Cogan
Matthew Coggins
F. Sessions Cole
Heather A. Colley
Heidi Cope
Rosario Corona
William J. Craigen
Andrew B. Crouse
Michael Cunningham
Precilla D’Souza
Hongzheng Dai
Surendra Dasari
Joie Davis
Jyoti G. Dayal
Esteban C. Dell'Angelica
Patricia Dickson
Katrina Dipple
Daniel Doherty
Naghmeh Dorrani
Argenia L. Doss
Emilie D. Douine
Dawn Earl
David J. Eckstein
Lisa T. Emrick
Christine M. Eng
Marni Falk
Elizabeth L. Fieg
Paul G. Fisher
Brent L. Fogel
Irman Forghani
William A. Gahl
Ian Glass
Bernadette Gochuico
Page C. Goddard
Rena A. Godfrey
Katie Golden-Grant
Alana Grajewski
Don Hadley
Sihoun Hahn
Meghan C. Halley
Rizwan Hamid
Kelly Hassey
Nichole Hayes
Frances High
Anne Hing
Fuki M. Hisama
Ingrid A. Holm
Jason Hom
Martha Horike-Pyne
Alden Huang
Sarah Hutchison

Wendy Introne
Rosario Isasi
Kosuke Izumi
Fariha Jamal
Gail P. Jarvik
Jeffrey Jarvik
Suman Jayadev
Orpa Jean-Marie
Vaidehi Jobanputra
Lefkothea Karaviti
Shamika Ketkar
Dana Kiley
Gonench Kilich
Shilpa N. Kobren
Isaac S. Kohane
Jennefer N. Kohler
Susan Korrick
Mary Kozuira
Deborah Krakow
Donna M. Krasnewich
Elijah Kravets
Seema R. Lalani
Byron Lam
Christina Lam
Brendan C. Lanpher
Ian R. Lanza
Kimberly LeBlanc
Brendan H. Lee
Roy Levitt
Richard A. Lewis
Pengfei Liu
Xue Zhong Liu
Nicola Longo
Sandra K. Loo
Joseph Loscalzo
Richard L. Maas
Ellen F. Macnamara
Calum A. MacRae
Valerie V. Maduro
AudreyStephannie Maghiro
Rachel Mahoney
May Christine V. Malicdan
Laura A. Mamounas
Teri A. Manolio
Rong Mao
Kenneth Maravilla
Ronit Marom
Gabor Marth
Beth A. Martin
Martin G. Martin
Julian A. Martínez-Agosto

Shruti Marwaha
Jacob McCauley
Allyn McConkie-Rosell
Alexa T. McCray
Elisabeth McGee
Heather Mefford
J. Lawrence Merritt
Matthew Might
Ghayda Mirzaa
Eva Morava
Paolo Moretti
John Mulvihill
Mariko Nakano-Okuno
Stanley F. Nelson
John H. Newman
Sarah K. Nicholas
Deborah Nickerson
Shirley Nieves-Rodriguez
Donna Novacic
Devin Oglesbee
James P. Orengo
Laura Pace
Stephen Pak
J. Carl Pallais
Christina G.S. Palmer
Jeanette C. Papp
Neil H. Parker
John A. Phillips III
Jennifer E. Posey
Lorraine Potocki
Barbara N. Pusey Swerdzewski
Aaron Quinlan
Deepak A. Rao
Anna Raper
Wendy Raskind
Genecee Renteria
Chloe M. Reuter
Lynette Rives
Amy K. Robertson
Lance H. Rodan
Jill A. Rosenfeld
Natalie Rosenwasser
Francis Rossignol
Maura Ruzhnikov
Ralph Sacco
Jacinda B. Sampson
Mario Saporta
Judy Schaechter
Timothy Schedl
Kelly Schoch
Daryl A. Scott

C. Ron Scott
Elaine Seto
Vandana Shashi
Jimann Shin
Edwin K. Silverman
Janet S. Sinsheimer
Kathy Sisco
Edward C. Smith
Kevin S. Smith
Lilianna Solnica-Krezel
Ben Solomon
Rebecca C. Spillmann
Joan M. Stoler
Kathleen Sullivan
Jennifer A. Sullivan
Angela Sun
Shirley Sutton
David A. Sweetser
Virginia Sybert
Holly K. Tabor
Queenie K.-G. Tan
Amelia L. M. Tan
Arjun Tarakad
Mustafa Tekin
Fred Telischi
Willa Thorson
Cynthia J. Tifft
Camilo Toro
Alyssa A. Tran
Rachel A. Ungar
Tiina K. Urv
Adeline Vanderver
Matt Velinder
Dave Viskochil
Tiphanie P. Vogel
Colleen E. Wahl
Melissa Walker
Stephanie Wallace
Nicole M. Walley
Jennifer Wambach
Jijun Wan
Lee-kai Wang
Michael F. Wangler
Patricia A. Ward
Daniel Wegner
Monika Weisz Hubshman
Mark Wener
Tara Wenger
Monte Westerfield
Matthew T. Wheeler
Jordan Whitlock

Lynne A. Wolfe
Kim Worley
Changrui Xiao
Shinya Yamamoto
John Yang
Zhe Zhang
Stephan Zuchner
